# Complete Androgen Insensitivity Syndrome in a Phenotypic Female: The Role of Frozen Section in Assisting Diagnosis

**DOI:** 10.7759/cureus.86431

**Published:** 2025-06-20

**Authors:** Vipul R Bhatt, Reena Tomar, Nikita Sharma, Deepti Goswami, Nita Khurana

**Affiliations:** 1 Pathology, Maulana Azad Medical College, Delhi, IND; 2 Gynecology, Maulana Azad Medical College, Delhi, IND

**Keywords:** amenorrhea, androgen insensitivity syndrome, frozen section, karyotyping, phenotypic female

## Abstract

Androgen insensitivity syndrome (AIS) is a rare disorder of sexual development. Patients present clinically with a varied phenotypic presentation depending on the residual androgen activity, which can be complete, partial, or mild. Karyotyping is helpful in the diagnosis of AIS. A suspicion of AIS should be considered in any female child presenting with inguinal swellings or labial edema. This report presents a case of this rare disorder in an 18-year-old unmarried girl who failed to achieve menarche. Tanner staging was performed, revealing a Tanner stage 4 for breast development and stage 2 for pubic hair. Clinical examination revealed bilateral inguinal swelling, clitoromegaly, and a blind-ending vagina. Radiological investigations additionally revealed the absence of the uterus and bilateral adnexal structures. Karyotyping was performed, which showed a 46,XY genotype. A surgical specimen was sent for a frozen section, which revealed fibro-collagenous tissue, blood vessels, epididymis, and hyalinized seminiferous tubules. Routine histopathological examination confirmed the absence of spermatogenesis and ruled out any neoplastic lesions. These findings were suggestive of complete AIS. This article highlights the pivotal role of frozen section in the diagnosis of such cases and emphasizes the importance of histopathological examination to rule out additional neoplastic etiology.

## Introduction

Androgen insensitivity syndrome (AIS), also known as testicular feminization, is a rare disorder of sexual development, with an incidence ranging from 1 in 20,000 to 1 in 100,000. Affected patients can exhibit a spectrum of phenotypes, ranging from phenotypic females to males with micropenis and infertility, depending on the level of residual androgen activity [1]. AIS can be classified into complete AIS (CAIS), partial AIS (PAIS), or mild AIS (MAIS), and diagnosis typically involves a multidisciplinary approach. The karyotype is usually 46,XY. Hormonal assays may not always be diagnostic. In CAIS, radiological examination reveals a blind-ending vagina and inguinal swellings [2]. Histopathological findings are essential not only for confirming the diagnosis by identifying the presence of immature testes in surgically excised inguinal swellings but also for ruling out any neoplastic lesions [3]. Therefore, the diagnosis, management, and follow-up of CAIS cases require a multidisciplinary approach [4]. Here, we present a case of CAIS in a young phenotypic female and discuss the diagnostic approach, emphasizing the significance of the frozen section, along with a review of the literature.

## Case presentation

An 18-year-old unmarried phenotypic female from North India presented to our hospital's gynecology OPD with the complaint of primary amenorrhea. She denied any history of cyclical abdominal pain, loss of smell, galactorrhea, headaches, blurred vision, hirsutism, trauma, drug intake, or heat/cold intolerance. There was also no history of tuberculosis, hypertension, or diabetes. She is the elder of two sisters, and her sister attained menarche at the age of 12 years. Her general condition was fair, with a height of 161 cm and a weight of 60 kg, yielding a BMI of 23.1 kg/m^2^. Tanner staging for secondary sexual characteristics revealed breast development at Tanner stage 4 (highest score 5), pubic hair at Tanner stage 2 (sparse, pre-sexual hair; highest score 5), and axillary hair at Wolfsdorf stage 1 (highest score 4). Pelvic examination revealed clitoromegaly, a blind-ending vaginal orifice, and bilateral inguinal swellings.

Serum hormonal profile showed the following: beta-HCG <2.39 mIU/mL (normal: non-pregnant female <5 mIU/mL); alpha-fetoprotein 3.6 IU/mL (normal: <10 IU/mL), CA125 7.6 U/mL (normal), total testosterone 32.3 nmol/L (elevated), follicle-stimulating hormone 14.10 mIU/mL (normal: 0.3-10 mIU/mL), luteinizing hormone 40.90 mIU/mL (normal: 5-25 IU/mL), serum lactate dehydrogenase 140 U/L (normal: 105-223 U/L), estradiol 56.10 pg/mL (normal: 30-400 pg/mL), and anti-Müllerian hormone 23.0 ng/mL (normal: 2.9 ng/mL).

**Figure 1:**
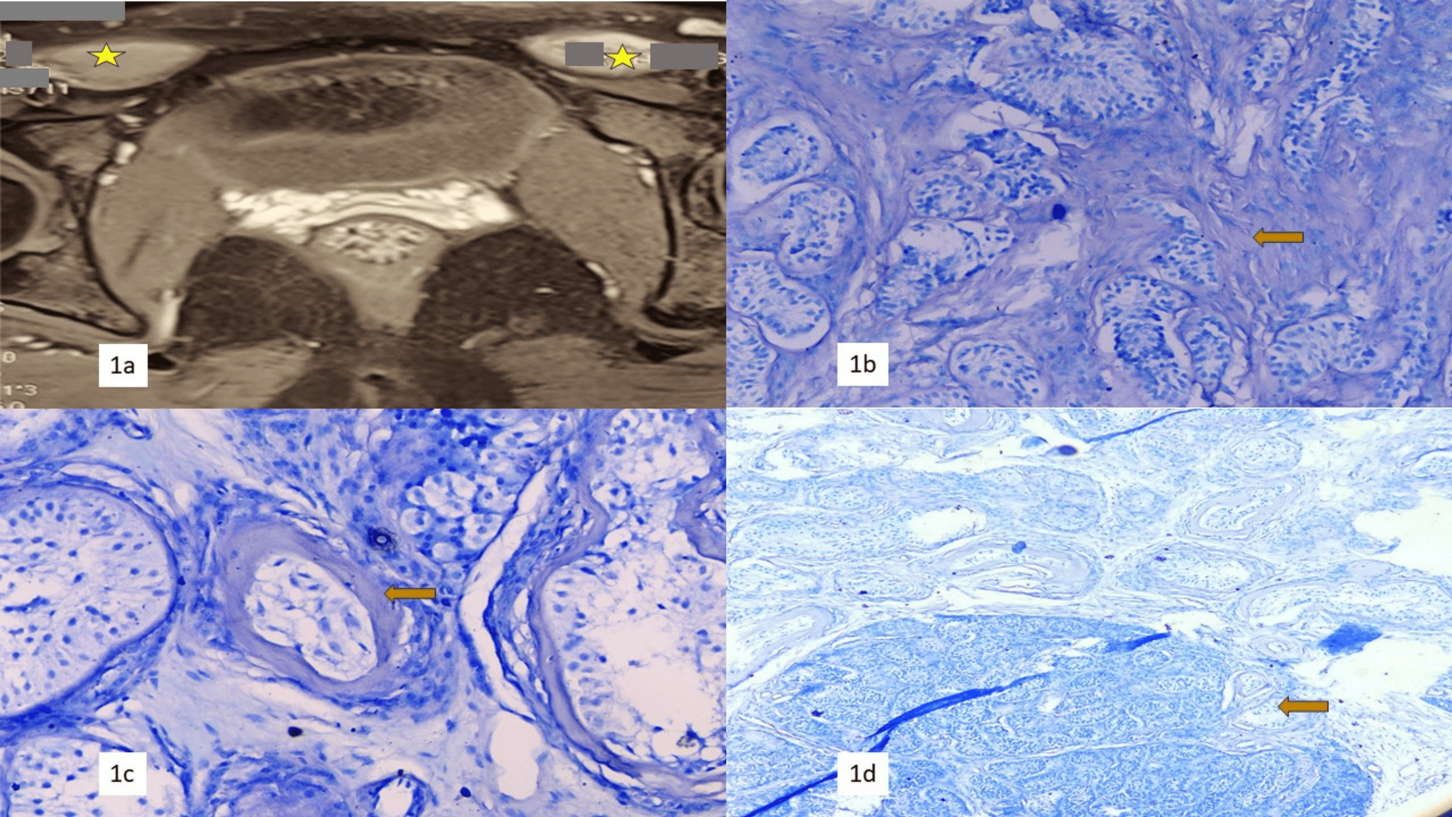
Frozen section and radiology (1a) CT scan image showing (highlighted by yellow asterisk) bilateral inguinal swellings. (1b) Cryosection showing seminiferous tubules with intervening fibrosis (200x toluidine blue). (1c) Cryosection showing hyalinized seminiferous tubules (400x toluidine blue). (1d) Cryosection showing a circumscribed lesion composed of variably thickened seminiferous tubules (100x toluidine blue).

Abdominal ultrasonography was performed, which revealed no obvious uterus or uterus-like structure behind the urinary bladder. No obvious ovaries or ovary-like structures were seen bilaterally. There was an oval, homogeneously hypoechoic lesion in the right inguinal canal, measuring 2.9 x 1.6 x 1.5 cm, with multiple ill-defined hypoechoic areas and internal vascularity. A similar lesion was identified in the left inguinal canal, measuring 2.9 x 1.6 x 3.4 cm (Figure 1a). MRI of the abdomen and pelvis showed similar lesions in both inguinal canals. The vagina measured 5.4 cm and appeared to end blindly. No uterus, cervix, or bilateral ovaries were visualized. A small subcentimeter cyst, likely a Müllerian duct cyst, was noted adjacent to the lesion. No other significant abnormalities were found in the abdomen. Subsequently, karyotyping was performed, which revealed a 46,XY karyotype.

**Figure 2:**
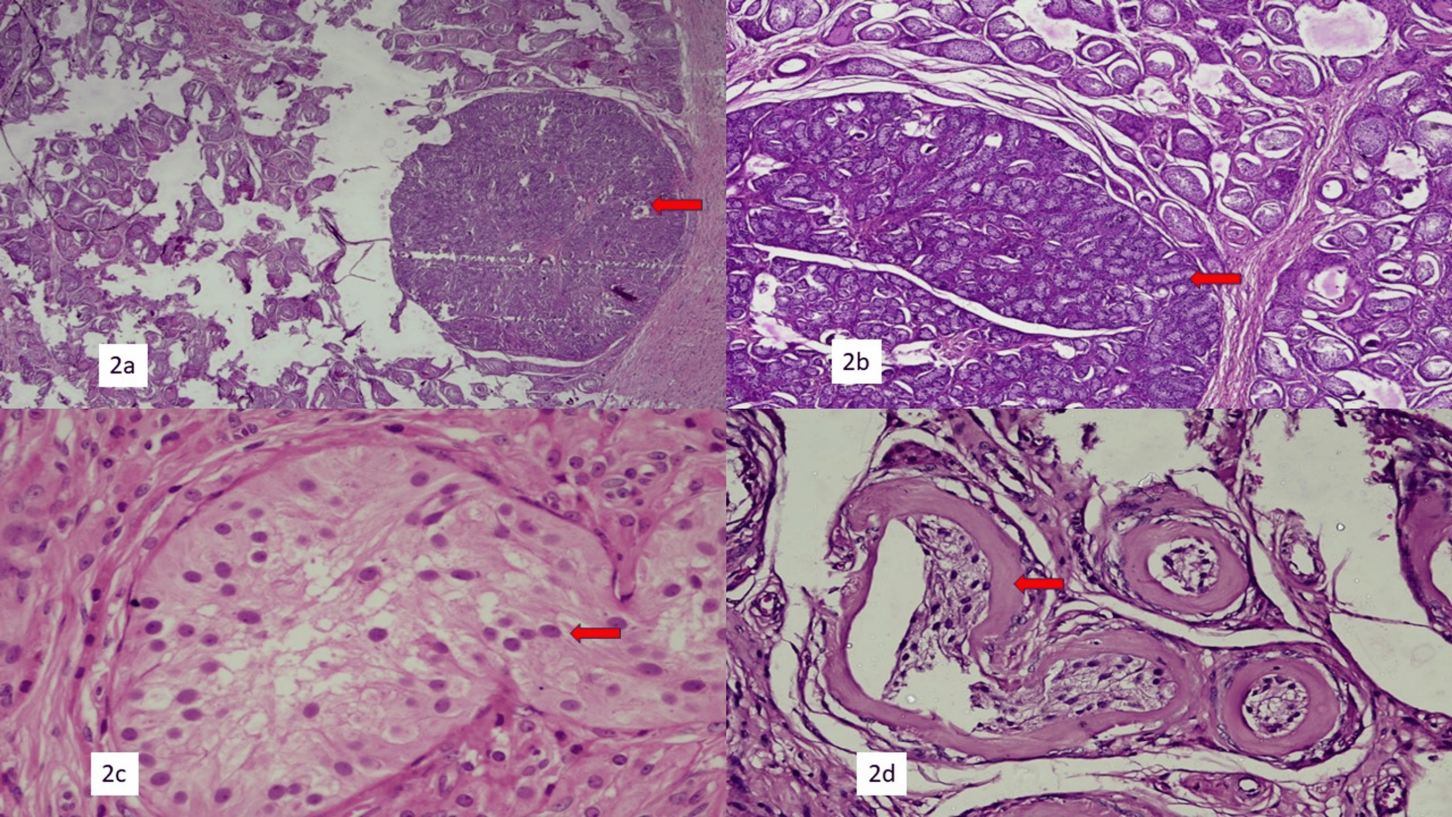
Histopathological images (2a) Seminiferous tubules with nodule formation (H&E, 100x). (2b) Hyalinized seminiferous tubules with Sertoli cell nodule formation (H&E, 200x). (2c) Seminiferous tubule with spermatogonia and the absence of mature sperms (H&E, 400x). (2d) Hyalinized seminiferous tubules (H&E, 200x).

Bilateral inguinal swellings were excised in toto and sent for frozen section and histopathological examination. Frozen sections from both the right and left inguinal swellings showed fibro-collagenous tissue, blood vessels, epididymis, and hyalinized seminiferous tubules, confirming the presence of male gonads (Figures 1b-1d). Histopathological examination revealed small hyalinized seminiferous tubules with variably thickened basement membranes in unencapsulated nodules, lined by Sertoli cells. Leydig cells were dispersed throughout the lesion. The Sertoli cells in many foci were arranged in a tubular or solid pattern, rimmed by a layer of fibroblasts and characterized by cuboidal to prismatic cells with pale eosinophilic to vacuolar, lipid-rich cytoplasm, and prominent nucleoli. The Leydig cells appeared as polygonal cells with distinct cell outlines and central round nuclei. No spermatogenesis was noted. There were no intratubular atypical cells, atypical mitoses, or stromal atypia (Figures 2a-2d).

A panel of immunohistochemical (IHC) markers, including calretinin, β-catenin, inhibin, alfa-fetoprotein, and placental alkaline phosphatase, was performed, all of which were negative. This helped to rule out Leydig cell tumors, Wilms tumor, sex cord-stromal tumors, yolk sac tumor, and germ cell tumors.

The findings were consistent with CAIS with Sertoli cell nodules. The patient is in regular follow-up and is receiving counselling.

## Discussion

AIS includes a group of mutations associated with resistance of target tissues, androgen receptor dysfunction, and impaired action of male hormones [4]. CAIS patients, who are phenotypically female, typically present with primary amenorrhea, infertility, and fully developed breasts due to the conversion of testosterone to estradiol [5]. Studies by Mukhopadhyay et al., Cruz et al., and Regadera et al. have highlighted the age of diagnosis as typically between 16 and 24 years, with clinical features similar to those observed in our case [2,4,6]. Radiological findings in studies by Mukhopadhyay et al., Nakamata et al., and Nezzo et al. were consistent with our findings of a blind-ending vagina, inguinal swellings, an absent uterus, and adnexal structures [2,7,8]. Karyotyping of affected individuals typically shows a 46,XY genotype, as was observed in our case. Histopathological examination of the lesion reveals the presence of immature testes. The seminiferous tubules are of varying sizes, peripherally rimmed by a dense basement membrane or hyalinization, with a band of fibroblastic proliferation. These tubules contain only Sertoli cells, with no germ cells present. The adjoining stroma shows Leydig cell proliferation, along with stromal edema, but without atypia. These findings were consistent with our case. No atypical cell proliferation or atypical mitosis was observed in our patient [3].

Vija et al. observed germ cells in two of their cases [9]. Rutgers and Rutgers noted spermatogonia in 28% of cases [10]. They also observed ovarian stroma in the majority of cases, and 9% of cases developed malignancies (two seminomas, one intratubular germ cell neoplasm with early invasion, and one malignant sex cord tumor). Siminas et al. identified bilateral para testicular leiomyoma with Sertoli cell adenoma in their research [3]. The ability of atypical germ cells to survive and the formation of further histopathological characteristics may be influenced by aberrant gonadal location and residual androgen receptor activity. However, the role of androgen insensitivity in increasing the risk of developing germ cell cancer remains debated. Since patient management strategies for individuals with complete androgen insensitivity have evolved, predicting the risk remains challenging [11]. The present case did not show any malignancy.

PAIS is difficult to diagnose and presents according to the level of androgen stimulation. Patients may exhibit a male phenotype, a female phenotype, or an indeterminate phenotype. They often present with ambiguous genitalia at birth, micropenis, hypospadias, bifid scrotum, clitoromegaly, gynecomastia, and otherwise normal testes, with normal or increased synthesis of testosterone and luteinizing hormone (LH). The present case did not show features of PAIS. A rare entity, MAIS, manifests in men as infertility, with other features such as gynecomastia or bulbar/spinal muscular atrophy (Kennedy’s disease). Diagnosis is aided by serum luteinizing hormone and testosterone levels [5].

## Conclusions

CAIS is rare, and karyotyping is essential for diagnosis. The diagnosis of CAIS requires a multidisciplinary approach, including clinical evaluation, radiological confirmation of a phenotypic female with primary amenorrhea, absence of Müllerian duct structures, and a karyotype that confirms a male genotype. Frozen section analysis was instrumental in establishing the diagnosis in this case. Histopathological examination remains the gold standard for confirming the diagnosis and ruling out malignancy. Comprehensive management of CAIS includes not only surgical excision, histopathological diagnosis, and evaluation of inguinally located testes, but also the assessment of characteristic histomorphology, such as germ cell aplasia, decreased testicular size, and tissue diameter. Additionally, post-treatment care should include psychiatric counseling, hormonal therapy, and gender reassignment surgery, if indicated.
